# Aggressive Basal Cell Carcinoma With Rib Metastasis: An Uncommon Presentation of a Common Skin Cancer

**DOI:** 10.7759/cureus.85762

**Published:** 2025-06-11

**Authors:** Junu Giri, Ammar Al Heyasat, Sandesh Neupane, Maida S Chaudhry, Indira Poojary

**Affiliations:** 1 Internal Medicine, Crestwood Medical Center, Huntsville, USA; 2 Internal Medicine, DHR Health Institute for Research and Development, Edinburg, USA

**Keywords:** basal cell carcinoma, cemiplimab, hedgehog pathway inhibitor, metastatic skin cancer, rib metastasis, vismodegib

## Abstract

Basal cell carcinoma (BCC) is the most common skin cancer, generally characterized by slow growth and minimal risk of metastasis. However, in rare cases, particularly those left untreated for extended periods, BCC can become deeply invasive and even metastatic. We report the case of a 72-year-old woman with a long-neglected ulcerating facial tumor that was ultimately diagnosed as BCC with extensive local invasion and biopsy-proven metastasis to the left anterior fifth rib. Imaging revealed destruction of the maxilla, orbit, and palate. The patient was managed with a multimodal approach, including hedgehog pathway inhibition with vismodegib, immune checkpoint blockade with cemiplimab, palliative radiation therapy, and ultimately extensive surgical resection. The coordinated therapeutic response led to clinical improvement and disease stabilization. This case underscores the potential aggressiveness of neglected BCC and reinforces the value of early detection and comprehensive, multidisciplinary management in advanced presentations.

## Introduction

Basal cell carcinoma (BCC) is the most common cutaneous malignancy in humans, comprising approximately 80% of non-melanoma skin cancers [1,2]. It primarily affects fair-skinned individuals and is closely linked to chronic ultraviolet exposure, particularly in older adults [2]. Although BCC is typically indolent and slow-growing, a small subset can become deeply invasive or, in rare cases, metastasize. The incidence of metastatic BCC (mBCC) is exceedingly low, estimated between 0.0028% and 0.55% of all BCC cases [3,4]. When it does occur, metastasis most commonly affects the regional lymph nodes, lungs, and bones [4,5]. Risk factors for advanced BCC or mBCC include tumor size greater than 3 cm, long duration, recurrent lesions, deep tissue involvement, aggressive histologic subtypes (e.g., basosquamous or basaloid), and immunosuppression [1-3]. Management of advanced cases has evolved significantly with the development of hedgehog pathway inhibitors (HHIs), such as vismodegib and sonidegib, which have shown meaningful clinical responses in both locally advanced and mBCC [1,6,7]. However, these agents are often limited by resistance or adverse effects, prompting the use of immunotherapy, most notably PD-1 inhibitors like cemiplimab, as an emerging second-line option [4,8,9]. We present a rare case of an elderly woman with a neglected, extensively invasive BCC of the face that ultimately metastasized to the rib. This case underscores the aggressive potential of untreated BCC and highlights the role of multimodal management incorporating systemic therapy, radiation, and surgery.

## Case presentation

A 72-year-old woman with a history of chronic anemia and anxiety was referred to the oncology clinic for evaluation of a progressively enlarging, ulcerated lesion on the right side of her face. According to the patient's daughter, the lesion had been slowly growing over the past 10 years, but the patient had not sought medical care. Over the preceding year, she experienced significant systemic symptoms, including approximately 40-50 pounds of unintentional weight loss. At the time of presentation, the lesion had become painful, with purulent discharge, frequent bleeding, and visible disfigurement.

On physical examination, the patient had a large, ulcerated, necrotic mass occupying the right cheek, upper lip, nasal bridge, and medial canthus, with extension into the oral cavity, upper palate, and possibly the orbit. A shave biopsy taken from the right lower lip margin confirmed the diagnosis of BCC. Initial laboratory workup, including complete blood count and basic metabolic panel, was unremarkable aside from known chronic anemia (Table 1).

**Table 1:** Initial laboratory evaluation demonstrating iron deficiency anemia, with additional liver and thyroid function tests. Reference ranges reflect institutional standards. MCV: mean corpuscular volume, TIBC: total iron-binding capacity, WBC: white blood cell, BUN: blood urea nitrogen, AST: aspartate aminotransferase, ALT: alanine transaminase, ALP: alkaline phosphatase.

Test	Result	Reference range
Hemoglobin (g/dL)	10.5	12.0-16.0
Hematocrit (%)	32.1	36-46
MCV (fL)	78	80-100
Serum iron (µg/dL)	35	60-170
TIBC (µg/dL)	420	240-450
Ferritin (ng/mL)	8	20-250
WBC (×10⁹/L)	6.4	4.0-10.5
Platelets (×10⁹/L)	280	150-400
Sodium (mmol/L)	138	135-145
Potassium (mmol/L)	4.2	3.5-5.1
BUN (mg/dL)	14	7-18
Creatinine (mg/dL)	0.9	0.6-1.3
AST (U/L)	22	10-40
ALT (U/L)	18	7-56
ALP (U/L)	88	44-147
Total bilirubin (mg/dL)	0.6	0.1-1.2
TSH (µIU/mL)	2.1	0.4-4.5
Glucose (mg/dL)	98	70-100 (fasting)

**Figure 1:**
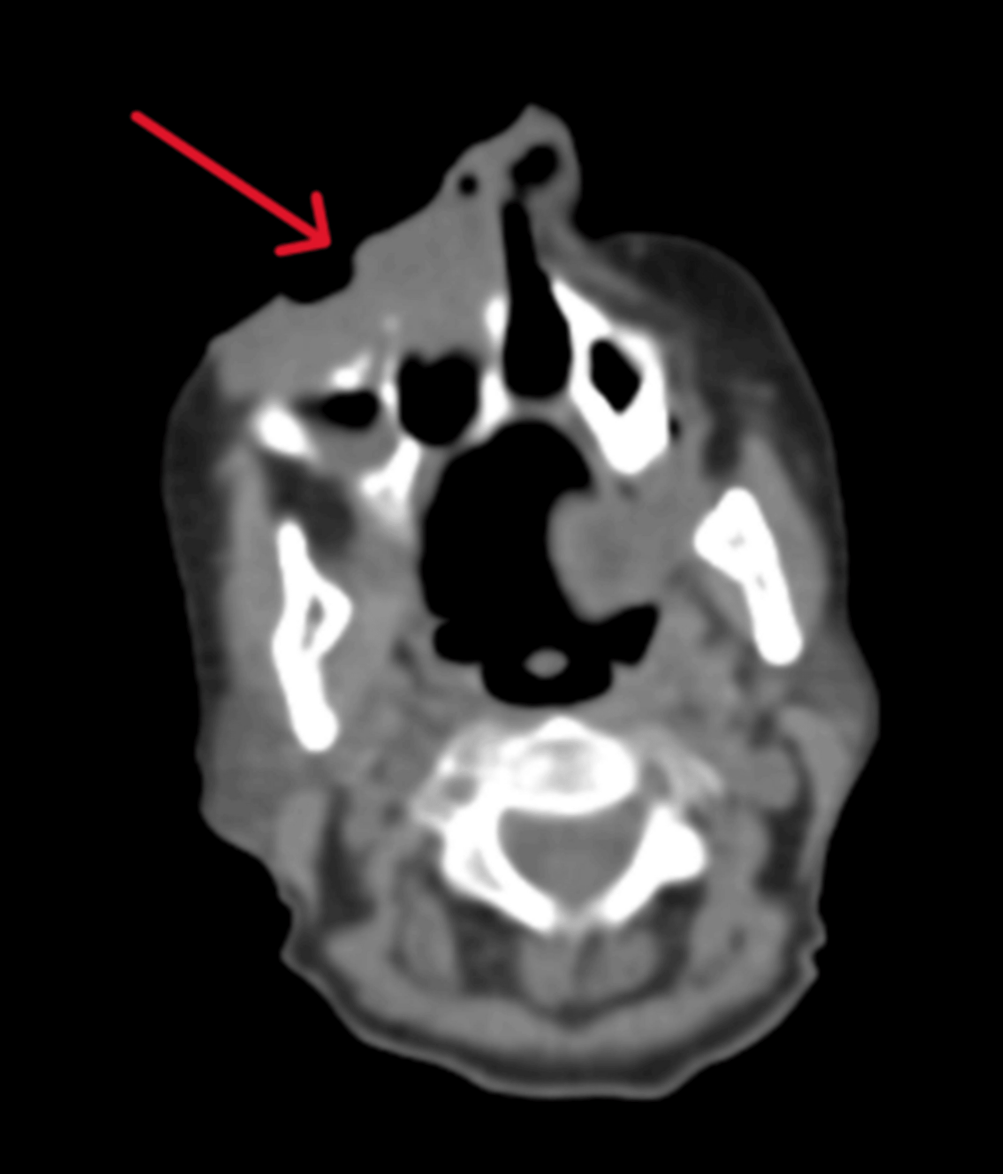
Axial CT scan of the head and neck showing extensive right facial basal cell carcinoma.

**Figure 2:**
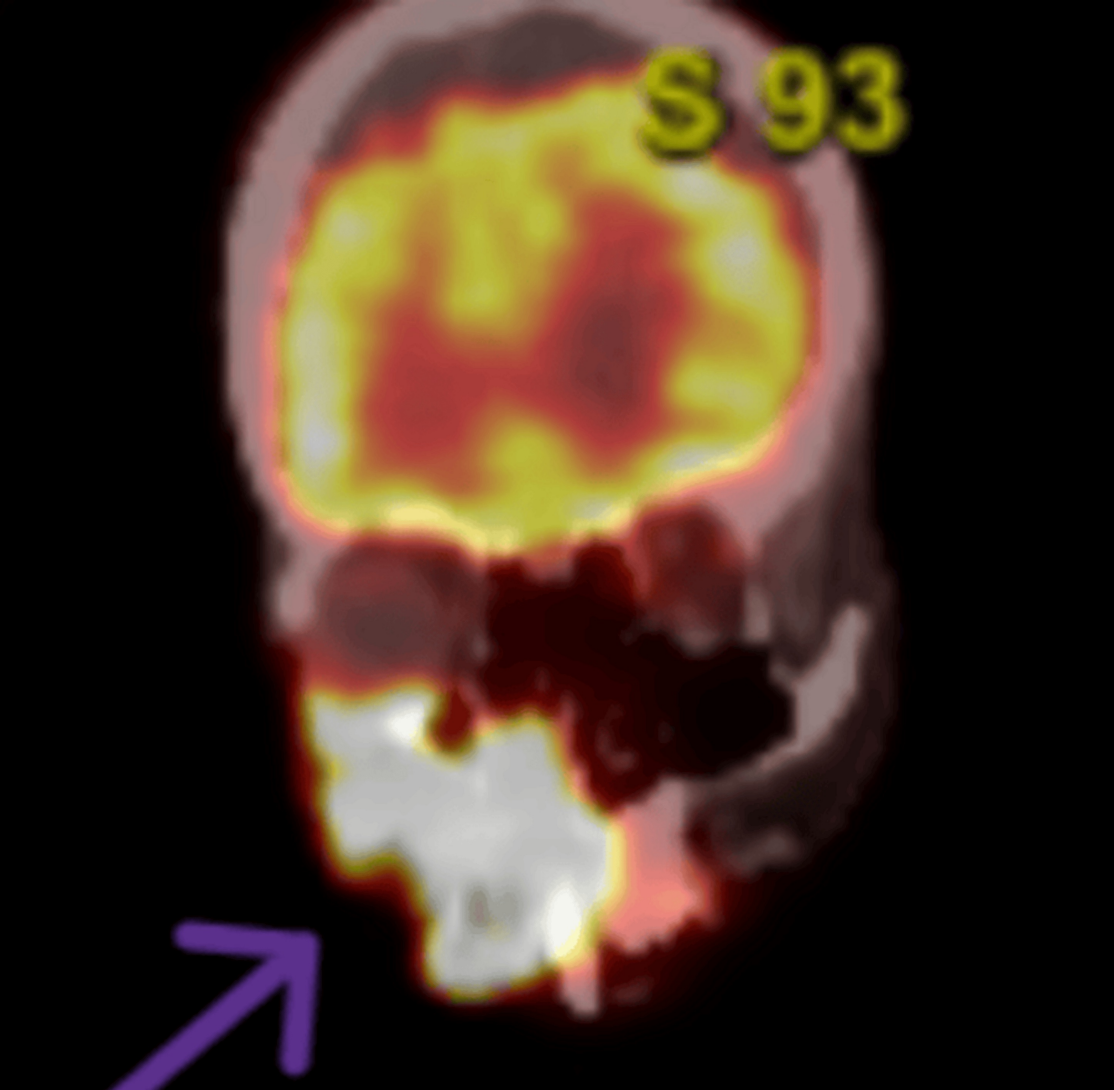
Coronal PET-CT image showing FDG-avid right facial tumor. PET-CT in the coronal view revealed a markedly fluorodeoxyglucose (FDG)-avid infiltrative mass in the right midface, consistent with aggressive basal cell carcinoma involving soft tissue and underlying bone.

**Figure 3:**
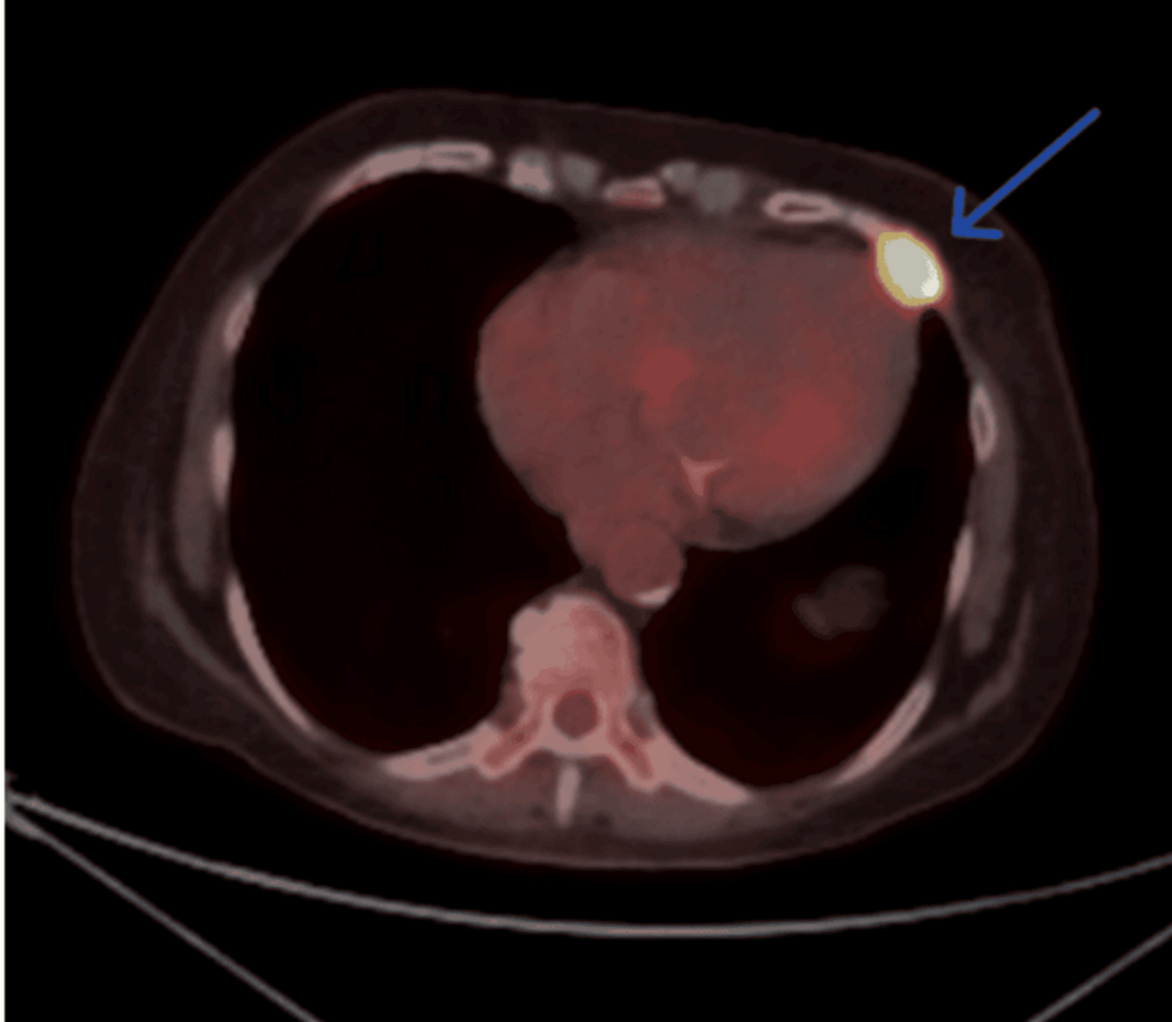
Axial PET-CT image showing FDG-avid lytic lesion in the left anterior fifth rib. Axial view of PET-CT revealed a fluorodeoxyglucose (FDG)-avid lytic lesion in the left anterior fifth rib with increased radiotracer uptake, consistent with osseous metastasis from the primary basal cell carcinoma.

**Figure 4:**
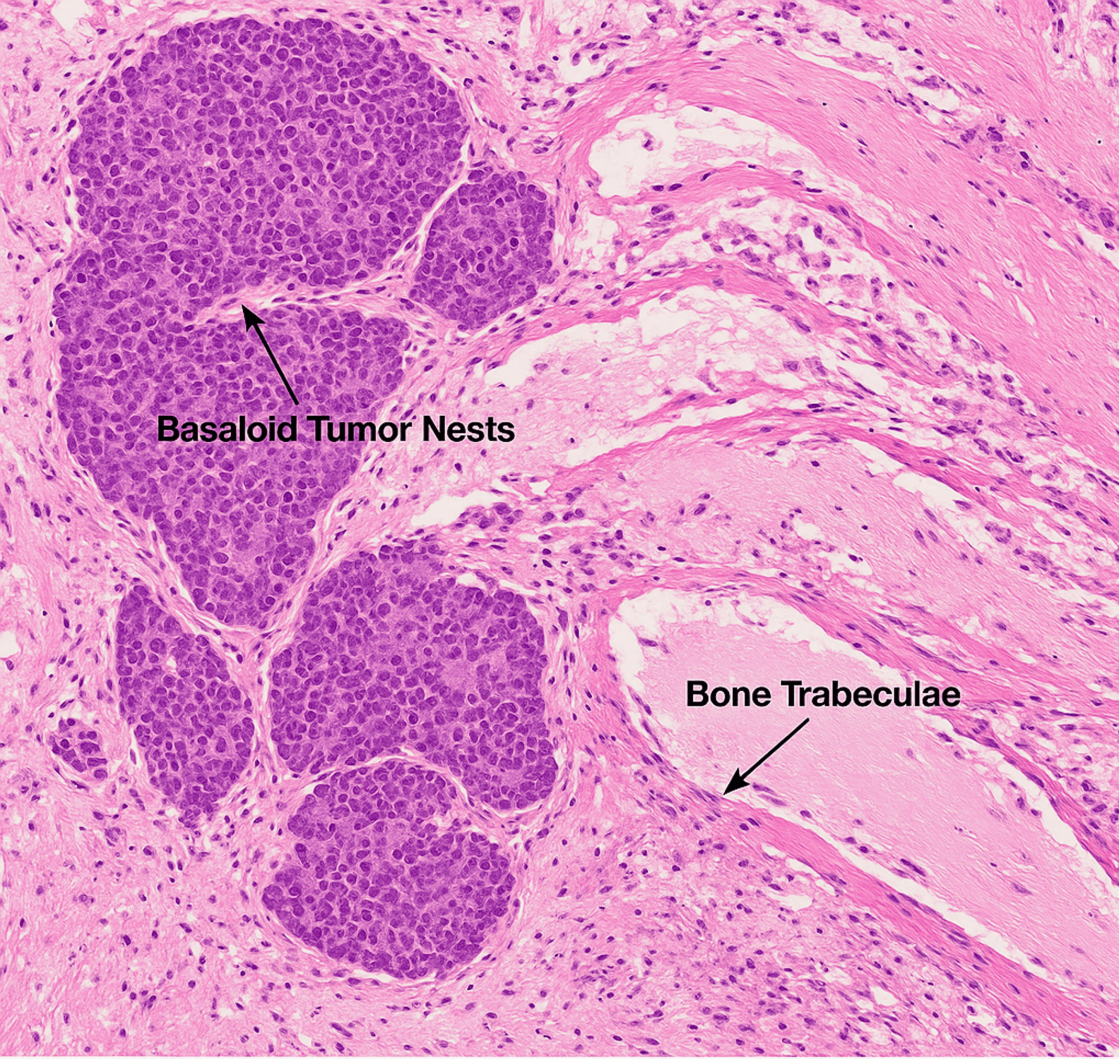
Histopathologic simulation of metastatic basaloid carcinoma involving rib bone tissue. Hematoxylin and eosin (H&E) staining demonstrated basaloid tumor nests (dark purple) with peripheral palisading infiltrating eosinophilic bone trabeculae. The surrounding stroma showed fibrous tissue and inflammatory infiltrates, consistent with osseous metastasis of basal cell carcinoma. The image was captured at 40x magnification.

To assess the full extent of the disease, a CT scan of the head and neck was obtained, which demonstrated a large, ulcerating mass centered in the right cheek. The lesion extended superiorly into the inferomedial orbital space, involving the right nasal cavity and inferiorly breaching the palate and oral cavity. Osseous destruction was evident, involving the hard palate, the right maxillary bone, the anterior right maxillary sinus, and the right alveolar process (Figure 1). Given these aggressive features, staging PET-CT imaging was performed, which revealed a markedly fluorodeoxyglucose (FDG)-avid right facial mass (Figure 2) and a lytic, FDG-avid lesion in the left anterior fifth rib, raising strong suspicion for metastatic disease (Figure 3). A CT-guided core needle biopsy of the rib lesion confirmed metastatic basaloid carcinoma consistent with BCC (Figure 4). The patient was staged as T4aN0M1, stage IV disease.

The patient was started on vismodegib 150 mg orally daily beginning on January 9, 2025. Due to the tumor’s friability and persistent bleeding, she also received palliative radiation therapy using a quad-shot regimen consisting of four doses of 350 cGy each, administered daily from January 3 to January 6, 2025, targeting the facial lesion. During follow-up, the lesion began to show clinical improvement, with reduced drainage and stabilization of the ulcerated surface.

However, concern persisted for progressive orbital invasion. In response, cemiplimab 350 mg intravenously every three weeks was added to the treatment regimen in early April 2025, approximately one month prior to the planned surgical intervention. The patient tolerated both systemic therapies well.

**Figure 5:**
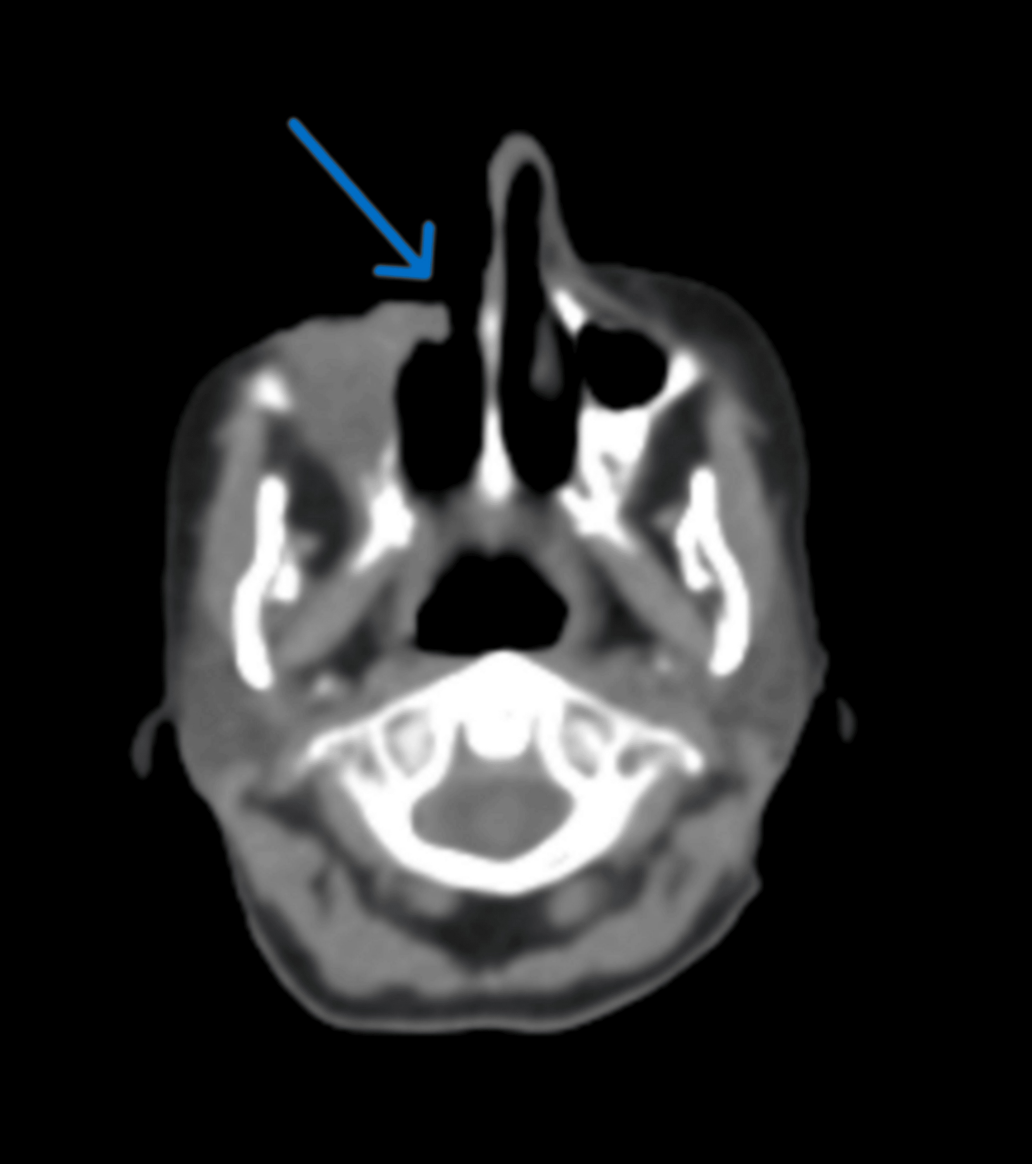
Axial post-treatment CT scan of the head and neck demonstrating tumor regression. Follow-up contrast-enhanced CT of the head and neck showed a significant decrease in the size of the previously noted right facial mass, approximately 45%-50% reduction in maximal tumor diameter. The tumor's extension into the orbit and oral cavity has regressed, indicating a favorable partial response to systemic therapy.

**Figure 6:**
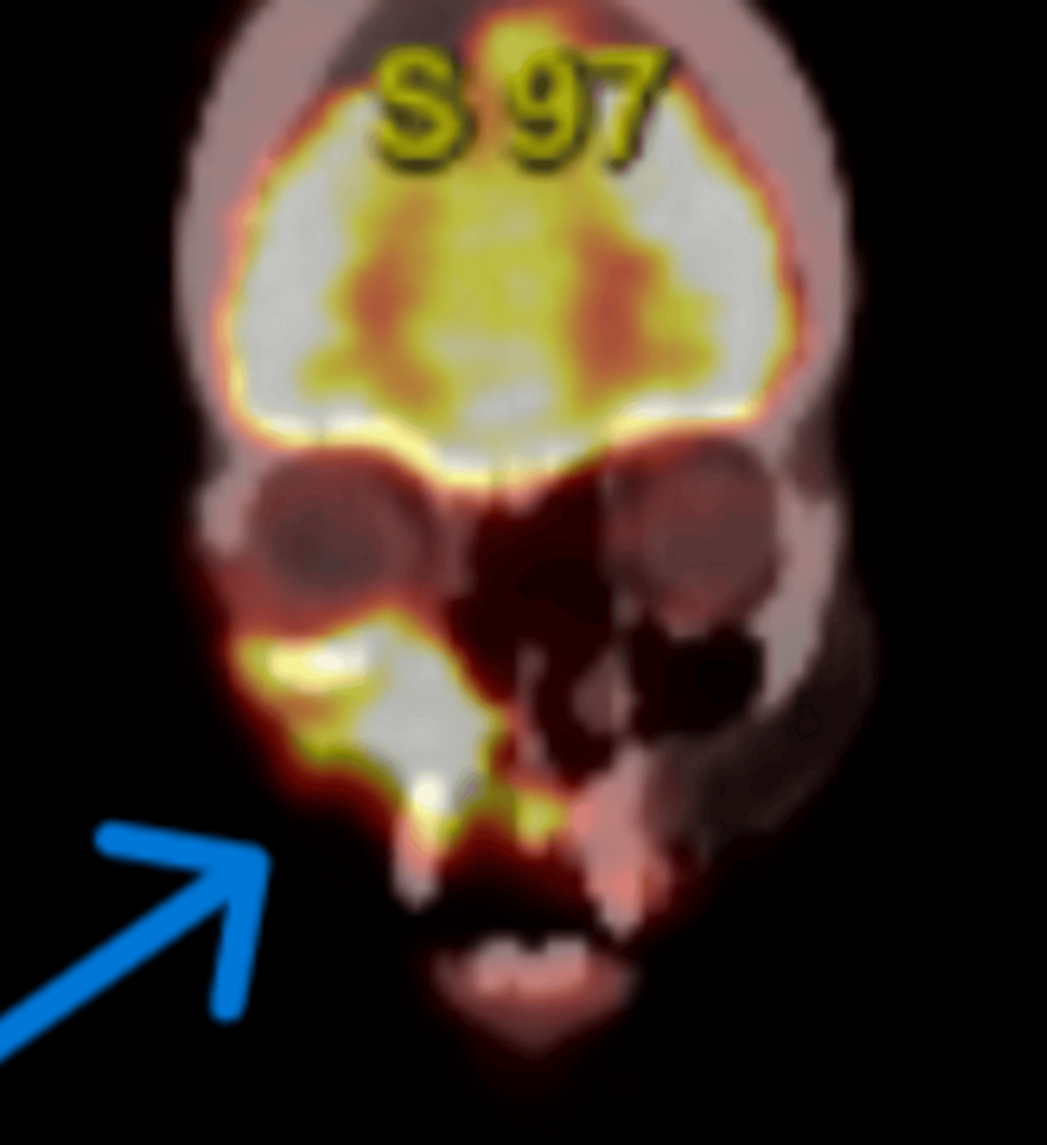
Coronal PET-CT image showing decreased metabolic activity of the facial tumor. Three-month follow-up PET-CT revealed a substantial reduction in fluorodeoxyglucose (FDG) uptake and volume of the right facial mass, consistent with metabolic and structural response to treatment.

**Figure 7:**
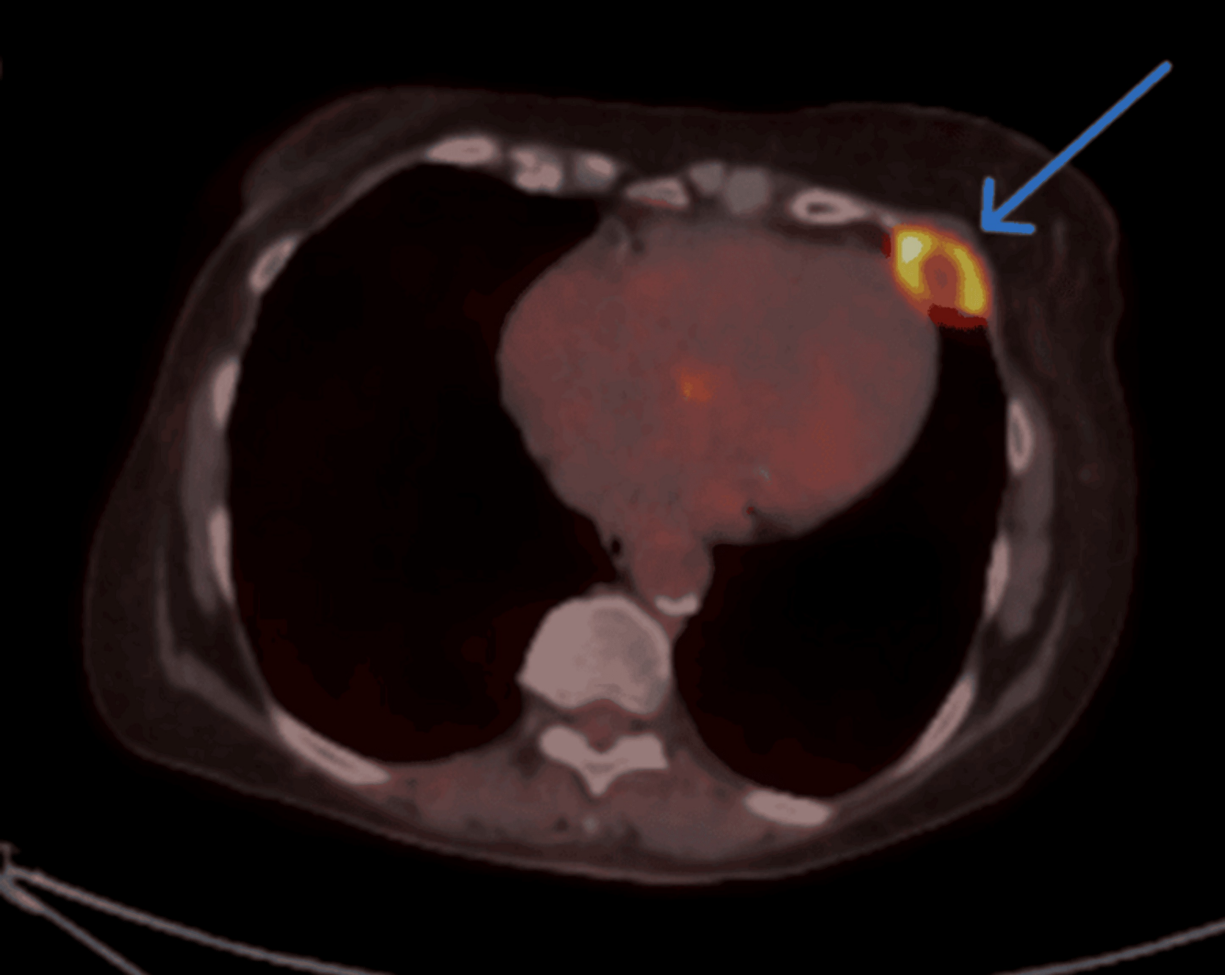
Axial PET-CT showing evolving necrosis in the metastatic rib lesion. Follow-up axial PET-CT of the chest demonstrated new areas of central necrosis within the fluorodeoxyglucose (FDG)-avid lytic lesion in the left anterior fifth rib, suggestive of a partial response to therapy.

In preparation for surgery, repeat imaging was obtained to reassess the disease status. A follow-up CT scan of the head and neck revealed a favorable treatment response, showing a significant decrease in the size of the mass centered on the right face (Figure 5). The infiltrative components into the orbit and oral cavity had also regressed. A repeat whole-body PET-CT performed three months after treatment initiation demonstrated a corresponding decrease in FDG activity and size of the right facial mass (Figure 6). Additionally, the FDG-avid lesion in the left anterior fifth rib exhibited new areas of central necrosis, interpreted as a partial response to therapy (Figure 7).

Encouraged by the radiographic findings and clinical stabilization, the multidisciplinary team proceeded with surgical resection on May 6, 2025. The patient underwent a right total maxillectomy with orbital exenteration, right rhinectomy, right modified radical neck dissection, and radical excision of the involved soft tissue. Surgery was extensive but uneventful. The patient recovered well postoperatively and resumed systemic therapy. Radiation therapy for the residual rib metastasis is being considered as part of her ongoing treatment plan.

## Discussion

Although BCC is the most frequently diagnosed cancer worldwide, it is typically associated with an excellent prognosis due to its slow growth and low metastatic potential [2,10]. Nonetheless, a small subset of tumors exhibit aggressive clinical behavior. mBCC remains an uncommon entity; however, when it occurs, it is most often associated with delayed diagnosis, extensive locoregional invasion, and high-risk histologic subtypes [1,4]. In our case, the patient delayed seeking care for nearly a decade, allowing the tumor to destroy the orbit, maxilla, and nasal structures and eventually metastasize to the left anterior fifth rib, confirmed on biopsy as mBCC.

Several factors have been shown to increase the risk of metastatic spread, including tumor size > 3 cm, head and neck location, perineural invasion, and basosquamous or basaloid histology [1,2,4,11]. Our patient’s tumor was not only large and long-standing but also located in a high-risk anatomic region, making the diagnosis of mBCC plausible and clinically consistent. While rare, bone metastasis, particularly to the ribs, has been reported in a limited number of cases, typically signaling an advanced and poor prognosis stage [4,5].

Historically, management of mBCC was limited to surgery and radiation, both of which have substantial limitations in inoperable or disseminated cases [3,12]. The introduction of HHIs has revolutionized systemic treatment. Vismodegib, a selective smoothened inhibitor, has demonstrated response rates of 30%-60% in advanced BCC, with durability of up to 26 months in some studies [6,7,13,14]. In our case, the patient responded favorably to vismodegib, which reduced the tumor burden and stabilized the disease. This allowed time for additional therapeutic planning and surgical coordination. Nevertheless, resistance and toxicity are frequent limitations of HHI therapy. In cases of progression or intolerance, immune checkpoint blockade has shown promise. Cemiplimab, a PD-1 inhibitor, has been approved for use in advanced BCC following HHI failure or intolerance and has demonstrated response rates of approximately 30% in this setting [4,8,9]. Our patient was treated with cemiplimab in combination with vismodegib prior to surgery, and radiologic imaging showed a favorable response, with necrosis in the rib lesion and tumor shrinkage on facial imaging. Surgical intervention remains the definitive approach in cases where tumor burden becomes resectable, particularly following systemic response [12]. Our patient underwent extensive resection, including maxillectomy, orbital exenteration, rhinectomy, and neck dissection. While this level of intervention is not typical for most BCC cases, it highlights the importance of aggressive, multidisciplinary management in exceptional presentations. Radiation therapy was also employed early in the treatment course to control bleeding and infection, underscoring its role in palliative and neoadjuvant settings [3,15]. The patient is now recovering postoperatively, with consideration of additional radiation to the rib metastasis. This case demonstrates the effectiveness of integrated therapy, including HHIs, immunotherapy, radiation, and surgery, in the management of mBCC. Ultimately, this case serves as a reminder that BCC is not universally benign. When neglected, it can be deeply destructive and even lethal. Timely diagnosis, patient education, and access to dermatologic care remain essential public health goals. In patients with advanced disease, personalized, multimodal strategies offer the best chance at long-term disease control and quality of life.

## Conclusions

Although BCC is typically considered an indolent skin malignancy, this case highlights its potential for aggressive local invasion and rare distant metastasis when neglected. Metastatic spread to the bone, such as the rib in this patient, is exceedingly uncommon and carries significant clinical implications. Early diagnosis and timely intervention remain critical in preventing such complications. This case underscores the importance of a multidisciplinary approach in managing advanced disease, integrating systemic therapy, radiation, and surgery. The combined use of vismodegib and cemiplimab demonstrated effective disease control, allowing for successful surgical resection. Clinicians should remain vigilant for atypical presentations of BCC, particularly in patients with long-standing, untreated lesions.

## References

[1] Wilson M, Johnson RP, Senft SC, Pan EY, Krakowski AC (2022). Advanced basal cell carcinoma: what dermatologists need to know about treatment. J Am Acad Dermatol.

[2] Nehal KS, Bichakjian CK (2018). Update on keratinocyte carcinomas. N Engl J Med.

[3] Schmults CD, Blitzblau R, Aasi SZ (2023). Basal cell skin cancer, version 2.2024, NCCN Clinical Practice Guidelines in Oncology. J Natl Compr Canc Netw.

[4] Nikanjam M, Cohen PR, Kato S, Sicklick JK, Kurzrock R (2018). Advanced basal cell cancer: concise review of molecular characteristics and novel targeted and immune therapeutics. Ann Oncol.

[5] Di Brizzi EV, Argenziano G, Brancaccio G, Scharf C, Ronchi A, Moscarella E (2023). The current clinical approach to difficult-to-treat basal cell carcinomas. Expert Rev Anticancer Ther.

[6] Frampton JE, Basset-Séguin N (2018). Vismodegib: a review in advanced basal cell carcinoma. Drugs.

[7] Basset-Seguin N, Hauschild A, Grob JJ (2015). Vismodegib in patients with advanced basal cell carcinoma (STEVIE): a pre-planned interim analysis of an international, open-label trial. Lancet Oncol.

[8] Absil G, Rorive A, Marchal N, Piret P, Nikkels AF (2025). Current treatment options for locally advanced and metastatic basal cell carcinoma. A narrative review. Expert Rev Anticancer Ther.

[9] Migden MR, Chang AL, Dirix L, Stratigos AJ, Lear JT (2018). Emerging trends in the treatment of advanced basal cell carcinoma. Cancer Treat Rev.

[10] Kim JY, Kozlow JH, Mittal B, Moyer J, Olencki T, Rodgers P (2018). Guidelines of care for the management of basal cell carcinoma. J Am Acad Dermatol.

[11] Kauvar AN, Cronin T Jr, Roenigk R, Hruza G, Bennett R (2015). Consensus for nonmelanoma skin cancer treatment: basal cell carcinoma, including a cost analysis of treatment methods. Dermatol Surg.

[12] Jacobsen AA, Aldahan AS, Hughes OB, Shah VV, Strasswimmer J (2016). Hedgehog pathway inhibitor therapy for locally advanced and metastatic basal cell carcinoma: a systematic review and pooled analysis of interventional studies. JAMA Dermatol.

[13] Słowińska M, Dudzisz-Śledź M, Sobczuk P (2022). Analysis of efficacy and safety of vismodegib therapy in patients with advanced basal cell carcinoma - real world multicenter cohort study. J Eur Acad Dermatol Venereol.

[14] Verkouteren BJ, Wakkee M, Reyners AK (2021). Eight years of experience with vismodegib for advanced and multiple basal cell carcinoma patients in the Netherlands: a retrospective cohort study. Br J Cancer.

[15] Seidl-Philipp M, Frischhut N, Höllweger N, Schmuth M, Nguyen VA (2021). Known and new facts on basal cell carcinoma. J Dtsch Dermatol Ges.

